# Folding the human proteome using BioNeMo: A fused dataset of structural models for machine learning purposes

**DOI:** 10.1038/s41597-024-03403-z

**Published:** 2024-06-06

**Authors:** Michael Hetmann, Lena Parigger, Hassan Sirelkhatim, Abraham Stern, Andreas Krassnigg, Karl Gruber, Georg Steinkellner, David Ruau, Christian C. Gruber

**Affiliations:** 1 Innophore, San Francisco, CA USA; 2 NVIDIA, Santa Clara, CA USA

**Keywords:** Protein folding, Protein structure predictions

## Abstract

Human proteins are crucial players in both health and disease. Understanding their molecular landscape is a central topic in biological research. Here, we present an extensive dataset of predicted protein structures for 42,042 distinct human proteins, including splicing variants, derived from the UniProt reference proteome UP000005640. To ensure high quality and comparability, the dataset was generated by combining state-of-the-art modeling-tools AlphaFold 2, OpenFold, and ESMFold, provided within NVIDIA’s BioNeMo platform, as well as homology modeling using Innophore’s CavitomiX platform. Our dataset is offered in both unedited and edited formats for diverse research requirements. The unedited version contains structures as generated by the different prediction methods, whereas the edited version contains refinements, including a dataset of structures without low prediction-confidence regions and structures in complex with predicted ligands based on homologs in the PDB. We are confident that this dataset represents the most comprehensive collection of human protein structures available today, facilitating diverse applications such as structure-based drug design and the prediction of protein function and interactions.

## Background & Summary

The human proteome is a highly interesting field to study, offering significant insights into the structural aspects of human biology. Exploring human proteins on a large-scale structural level is of great importance, especially when it comes to drug development and off-target search, where structural information plays a crucial role. As of April 2023, UniProt^1^ provides a comprehensive collection of 81,671 protein sequences within the reference proteome UP000005640, representing potential proteins and their splicing variants for 19,357 distinct (nonsynonymous) human genes with only about half of these sequences have been experimentally confirmed on the protein level.

Previously, EBI and DeepMind^2,3^ made great efforts to model the human proteome with AlphaFold 2, providing structural models for 74.32% of the human reference proteome UP000005640.

Focusing on full length proteins whose existence has been proven on protein level and disregarding protein fragments, the reference proteome yields 42,158 proteins and their splicing variants for 16,303 distinct genes. The AlphaFold database already includes models for 77.76% ( = 32,782 structures) of this subset of protein sequences. At the time this manuscript was submitted, 498 of these models contained truncated sequences or point mutations compared to the respective sequences in the reference proteome.

With the dataset we present here, we aim to extend the structural information of the human proteome, and provide atomistic 3D-models for 99.72% ( = 42,042 structures) of the above-mentioned subset of human protein sequences. To increase structural diversity, and thus enhance quality and comparability, we provide structures generated with three state-of-the-art AI-guided structure prediction tools AlphaFold 2^2^ (with 32,284 available models retrieved from the AlphaFold database), OpenFold^4^, and ESMFold^5^ (provided within NVIDIA’s BioNeMo platform [https://www.nvidia.com/en-us/gpu-cloud/bionemo/]), as well as homology modeling using Innophores CavitomiX platform^6–8^.

We recommend keeping the structural diversity as presented herein in mind, as different modeling tools result in significant variation in structural compositions. These can equally significantly influence the outcome of structural bioinformatic pipelines like virtual docking, molecular-dynamics simulations, and other downstream processes. Even though the performance of the different modeling tools is expected to vary for specific cases, a combination of models generated in different ways is expected to enhance both the quality and representative scope of this dataset. Structural diversity might also present native-like conformational states. For instance, previous work by Alamo *et al*. demonstrated that reducing the depth of the input multiple sequence alignments in AlphaFold modeling resulted in conformations spanning the range between two experimental structures^9^. Further, if certain regions of the protein are modeled in a similar way using different methods, the general modeling confidence of this region can be considered higher than for regions that show significantly different conformations when modeled using different algorithms.

Especially when considering applications of the provided models in drug discovery or in the screening for potential off-target binding sites of therapeutics, analyses of potential binding sites for small molecules are of high interest. In the homology models we provide, cofactors and ligands are automatically included based on the template used for structural modeling. To increase information regarding potential binders in the AI-predicted models, which initially do not contain small molecules, we provide a separate dataset where ligands are included in these models, if they show sufficient structural similarity to the respective homology models. It is worth noting that DeepMind recently announced a new version of AlphaFold, with enhanced accuracy and modeling capabilities which go beyond protein structures and will include small molecules, DNA/RNA as well as post-translational modification, which will allow to include ligands and cofactors already during the modeling process^10^.

This could be of particular importance in cases where the homology model was created for only part of the protein sequence (which is the case when there is no crystal structure available as a template for the full sequence). As AI-generated models span the whole protein sequence, they might indicate additional interactions between the ligand and residues missing in the homology models. We further encourage interested researchers to explore the full potential of binding sites (independent from the availability of crystal structures) in the provided models using the CavitomiX PyMOL plugin, which is described in more detail in the section Usage Notes.

Opposed to homology models, which are generated for protein regions which show suitable similarity to structures deposited in the PDB, the AI-predicted models provided herein represent the structure of the full protein sequence in every case. Depending on the availability of homologous sequences, AI-predicted models may contain regions which were modeled with low confidence. As such regions might cause various problems in structural analysis or downstream processing, we additionally provide a refined dataset of structures, in which these low-confidence regions were removed. While the structural integrity of a protein might be distorted upon excision of such regions, there are applications which benefit from the removal. For example, in the analysis of ligand binding sites, low-confidence regions, e.g., a transmembrane region, which mostly appear as unstructured loops in the AI generated models, might block the binding site cavity. In nature, such regions would be integrated into the membrane and would thus be prohibited from blocking this binding site. On the other hand, when studying protein dynamics, for example, missing parts in a structure can be a significant disadvantage. To facilitate the usefulness of this data for different applications, we provide the structures in an unrefined (full-length protein models) and refined (without unstructured low-confidence regions) collection.

**Fig. 1:**
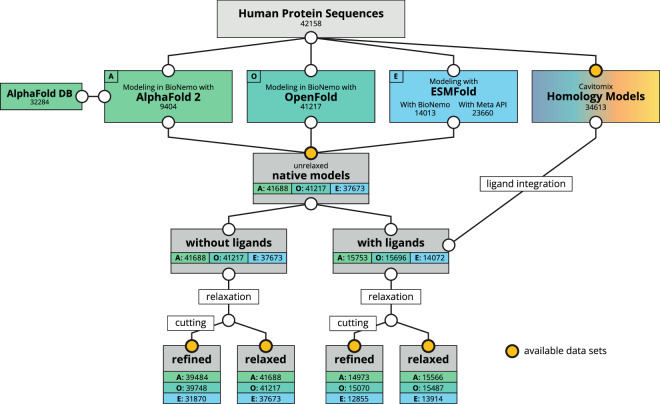
Flowchart illustrating the generation method and structure of the dataset. The numbers show how many of the respective models are provided in the respective data records. Circles filled with a yellow color indicate which data records are available for download. “Relaxation” refers to energy minimization with the Amber03 force field and “cutting” refers to the removal of low confidence regions. A: AlphaFold 2; O: OpenFold; E: ESMFold.

In total, we provide a collection of 122,907 novel protein structures. Together with available structures from the AlphaFold database, we refined our models with energy minimization, removal of low-confidence regions and including potentially binding ligands. Finally, we provide both native and refined data records for download. An overview of the generation workflow and structure of the data is depicted in Fig. 1.

The resulting dataset is, to the best of our knowledge, the most comprehensive structural dataset for the human proteome up to date. We believe that it will be useful for the community and can be applied in various structural-bioinformatic and machine-learning processes. While we have carefully curated the dataset, we still need to emphasize that the provided structures are computational predictions, and may not necessarily represent the natural protein structure, especially for protein sequences with little homologous information in existing databases. A comprehensive table including valuable information and statistics on each structural model is included as a separate data record and should allow readers to critically assess each model’s quality.

## Methods

### Retrieval of protein sequences

Protein sequences were obtained in April 2023 from the UniProt Database^1^ using the search filter “UP000005640 AND (existence:1) NOT (fragment:true)”. The resulting 42,158 sequences comprise proteins confirmed to exist at protein level within the human reference proteome UP000005640, excluding protein fragments.

### Structural modeling

Homology modeling was performed on the full set of 42,158 human protein sequences using the CavitomiX platform^6–8^ with integrated YASARA^11^. Six PSI-BLAST iterations, a maximal Expect value of 0.5 and a maximum of two alignment variations per template were used, with 50 conformations tried per loop. We considered one template per model, a maximum oligomerization state of 25, and omitted modeling terminal loop residues, which protrude beyond the sequence alignment. For 7,545 sequences, no suitable template for modeling was found, therefore 34,613 homology models are included in the dataset (Fig. 1).

AI-guided structure prediction was performed on a selection of 41,688 protein sequences. These sequences were filtered for a length of 10–2,700 amino acids and do not contain unknown characters (U, O, X, J, B, Z). Regarding AlphaFold 2, 32,284 structural models were already available in, and therefore downloaded from, the AlphaFold database^3^. The remaining 9,404 sequences were modeled with AlphaFold 2 using NVIDIA’s BioNeMo platform. In total 41,688 AlphaFold 2 models are included in the dataset (Fig. 1). Due to technical constraints, OpenFold comprises an upper limit of 2,000 amino acids for the sequence length of models. 41,217 OpenFold models were predicted using NVIDIA’s BioNeMo platform. The upper limit for sequence length for ESMFold within the BioNeMo platform is 1,024 amino acids, while it is 400 amino acids for Metas ESM-API^5^. Therefore, 23,660 sequences with 10–400 amino acids were created using the ESM-API, while the remaining 14,013 sequences were modeled with ESMFold embedded in NVIDIA’s BioNeMo platform. In total 37,673 ESMFold models were built (Fig. 1).

All AI-predicted models were initially generated without subsequent relaxation ( = energy minimization). Energy minimization and further downstream processes, like the inclusion of ligands, were performed during the refinement of structures, which is explained in detail below.

### Incorporation of ligands into AI-generated models

**Fig. 2:**
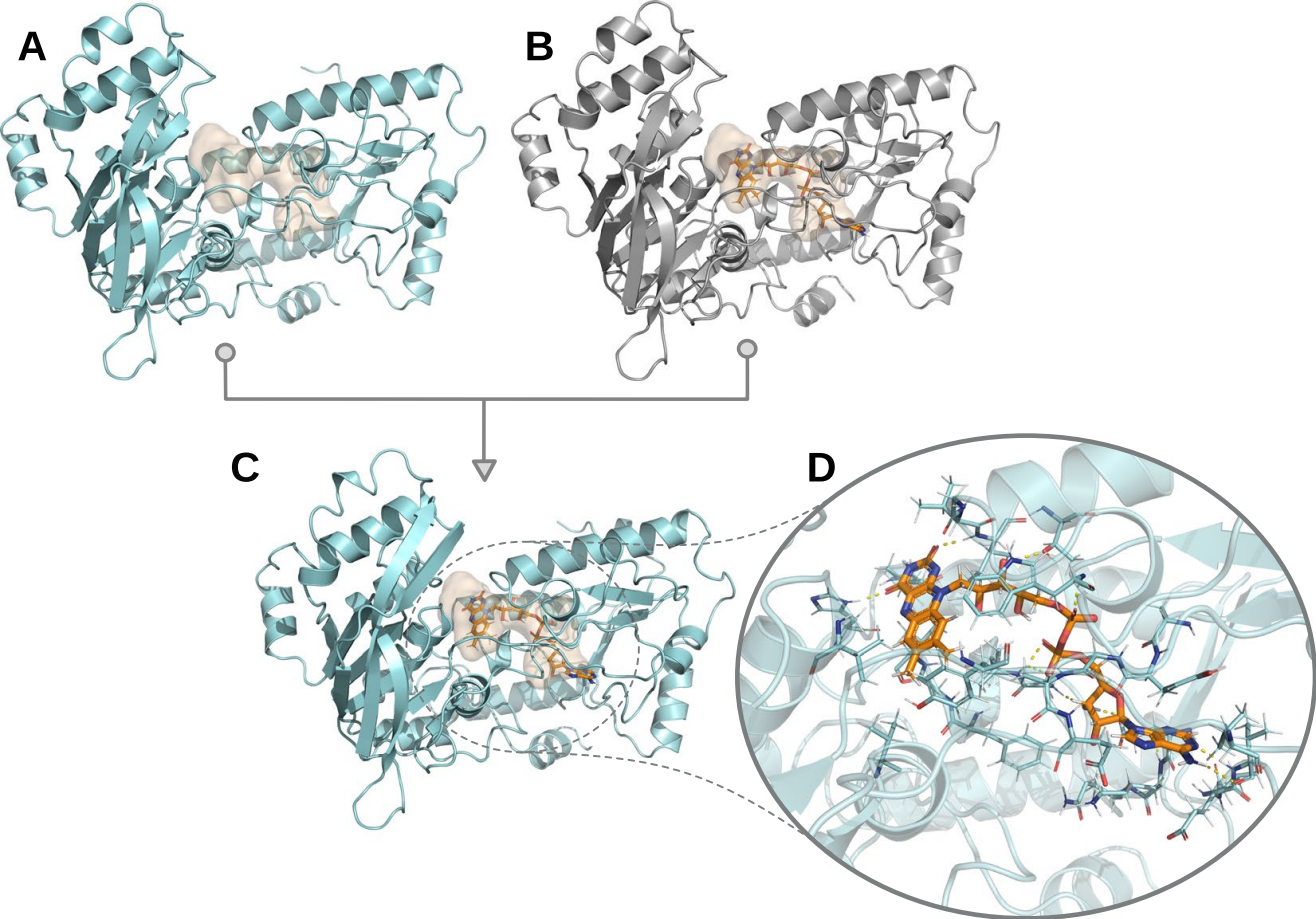
Example ligand incorporation. The ligand-binding cavity was calculated using the CavitomiX PyMOL plugin (https://innophore.com/cavitomix/) and is displayed as an orange transparent surface. (**A**) OpenFold model for sequence A0A0A0MTN9 (UniProt identifier). (**B**) Homology model for sequence A0A0A0MTN9, containing flavin adenine dinucleotide (FAD). (**C**) Refined OpenFold model from A), with the cofactor FAD (orange sticks) incorporated based on its 3D-conformation in the homology model. (**D**) Zoom on the residues within 3 Å around FAD, shown as turquoise sticks. Polar interactions between FAD and amino acids are displayed as yellow dashed lines.

Models generated with AI-based methods initially do not contain any ligands. To include the information of potential binding sites for small molecules, which might activate, inhibit, or modulate the proteins function, we incorporated ligands subsequent to structural modeling (*e.g*., see Fig. 2). To ensure comparability between the ligand interaction sites of proteins modeled with the different structure prediction tools used in this study, we chose to transfer ligands from the homology models to the AI-generated models. In short, if, upon structural alignment, ligand-binding sites in the homology models showed sufficient structural similarity to regions in the AI-generated models, ligands were copied to these models, which were energy minimized and screened for clashes afterwards.

First, the AI-predicted model, together with, if applicable, the homology model built from the same sequence, were parsed with Biopython^12^. If ligands were present in the homology model, a sequence alignment of the AI-predicted model with each chain of the homology model was performed, to identify the best matching chain. If two chains matched equally well, the one containing the most ligands was selected.

We then used PyMOL^13^ to align the 3D structure of the best-matching chain, together with all the ligands within the homology model, to the AI-predicted model in different ways, iterating through each ligand contained in the respective chain in the homology model: First, it was checked if the ligand is within 5 Å of any atom in the homology model to ensure that physical interaction between ligand and model is possible. Second, different alignment strategies were iterated (selecting residues within 5–15 Å of the ligand, and aligning with alternating algorithms “align” and “super” in PyMOL). The conformation that leads to the lowest RMSD in the region around the ligand between the homology model and the AI-predicted model was selected. If the defined maximum RMSD of 7 Å was reached, the ligand was copied to the AI-predicted model, otherwise the incorporation was omitted. DNA and amino acids labeled as “HETATM” in the PDB file were excluded from the ligand incorporation protocol.

After inclusion of ligands, an energy minimization was performed and low confidence regions were removed, as explained in more detail below. In cases where the energy minimization did not suffice to release clashes between the ligand and model, the specific ligand was deleted from the PDB file. After removing low confidence regions from the ligand-containing AI-predicted models, ligands that were no longer within 5 Å of any atom in the model were discarded.

### Energy minimization of AI-predicted models

Since the models built with AI-driven tools were initially generated without subsequent energy minimization, energy minimization was performed as an additional step after the modeling process, using the Amber03 force field in YASARA^11^. Energy minimization was performed on both the native and ligand-incorporated models before removing low confidence regions. Notably, BioNeMo also provides the option to perform energy minimization after structure inference.

### Removal of low confidence parts from AI-generated models

**Fig. 3:**
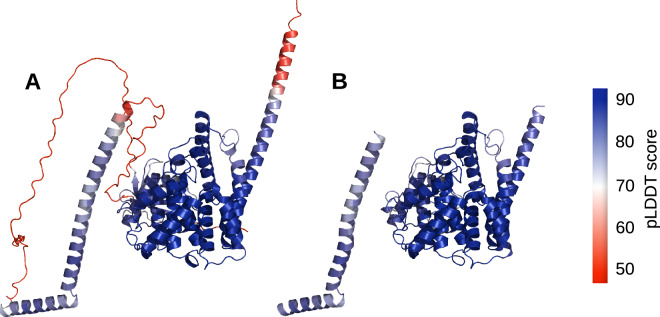
Example removal of low confidence regions. (**A**) Native AlphaFold 2 model for sequence O76083 (UniProt identifier). (**B**) Refined model for O76083, after energy minimization and removal of low confidence regions. Structures are colored per residue by their pLDDT score (see color bar).

ESMFold, OpenFold, and AlphaFold 2 structures can contain regions which were modeled with low confidence, *e.g*., due to low sequence coverage, leading to little evolutionary or structural information which can be connected to the sequence. Depending on the application, such low confidence regions might negatively influence downstream analyses, e.g., molecular docking, of the respective models. Therefore, we decided to provide an additional dataset in which these low-confident regions were removed from the structures (*e.g*., see Fig. 3).

A valuable metric to describe the reliability of structural models, known as the predicted local distance difference test (pLDDT)^14^ score, was considered in the removal of low confidence regions. For each residue within a model, we calculated the average pLDDT score within a region of +/− three amino acids before and after the respective residue. If this average pLDDT score falls below 70, the residue is flagged for removal. Employing this averaging approach over a range of amino acids helps to avoid the selective removal of individual residues that only slightly dip below the pLDDT threshold of 70.

In certain scenarios, short amino acid chains may show an average pLDDT score meeting the threshold but are geometrically quite far from the typically well-defined (high pLDDT score) core of the protein. To address this, we applied an additional criterion: Short amino-acid chains were removed if they are located more than 10 Å away from the protein core or more than 5 Å away from another short amino-acid chain that itself is within 10 Å of the protein core. After the refinement, the dataset was cured from structures containing less than ten amino-acid residues, and (for the ligand-containing models) from ligands not being within 5 Å of any atom in the respective models.

Notably, we chose the pLDDT score for quality assessment rather than the predicted aligned error (PAE), which is another quality parameter for structure prediction confidence and gives a distance error for every residue-pair. Our decision was based on the fact that the pLDDT is a universal value which is presented by AlphaFold, OpenFold and ESMFold, whereas PAE is only provided for AlphaFold models. We are confident that the pLDDT is a sufficient parameter for refining structures by removing low-confidence regions.

## Data Records

Next to protein information and statistics, including quality parameters, pairwise RMSD calculations and Ramachandran plots, we provide the structural dataset in different formats, representing progressive refinement stages of the models. The overview of the whole dataset and the steps taken to create the individual data records are illustrated in Fig. 1; the naming scheme of the data records containing the models is identical to the naming in Fig. 1. Furthermore, we provide Table 1 for clarity regarding the organization of deposited PDB files. All data records can be downloaded individually as compressed folders from figshare^15^. The PDB files contain headers and titles, showing the UniProt accession numbers and the protein descriptions, respectively.

**Table 1:** Organization of deposited PDB files.

Data record	Subfolders	Filenames
Homology models	—	HM_[accession].pdb
Native AI-predicted models	[model_type]_models	[model_type]_[accession].pdb
Relaxed AI-predicted models	Relaxed_[model_type]_models	relaxed_[model_type]_[accession].pdb
Refined AI-predicted models	Refined_[model_type]_models	refined_[model_type]_[accession].pdb
Relaxed AI-predicted models with ligands	Relaxed_[model_type]_models_with_ligands	ligand_integrated_relaxed_[model_type]_[accession].pdb
Refined AI-predicted models with ligands	Refined_[model_type]_models_with_ligands	ligand_integrated_refined_[model_type]_[accession].pdb

The term [model_type] refers to either of the three tools AlphaFold 2, OpenFold, or ESMFold and [accession] refers to the protein’s UniProt accession number.

For protein models generated with AI-guided tools AlphaFold 2, OpenFold, and ESMFold, the pLDDT score is given in the B-factor column within the PDB files. It serves as a per-residue confidence measure, providing crucial insights into the reliability of the protein models and specific regions therein. It is important to note that pLDDT values exceeding 90 are indicative of a high level of confidence in the accuracy of the structural predictions, whereas those of below 70 is considered as low confidence and thus the respective structural regions should be considered less reliable.

The structural quality of homology models can be determined by the z-score (optimal: >0, satisfactory: −2 - 0; poor quality: <−2). Further quality parameters are the sequence identity, similarity and coverage of the input sequence to the sequence of the template.

### Protein information and statistics

For quality control, we provide Ramachandran plots generated with Biopython^12^, employing the package Bio.PDB, as well as the results from a pairwise alignment of models in the form of CSV files for the native and refined models. Ramachandran plots are available for native, relaxed and refined versions of the models.

Further, a compressed CSV file (protein_information_and_statistics.csv) contains valuable information regarding the protein sequences and models. The file comprises 42,158 entries, each corresponding to a protein sequence considered in the modeling process. It includes UniProt accession codes, gene names (and synonyms), amino-acid sequences, details about the model type available for each protein (AlphaFold 2 or downloaded from the AlphaFold database, OpenFold, ESMFold, homology model), and, if applicable, the ligands they include. Additionally, the file provides detailed information about each model’s secondary structure as well as a statistical analysis of quality parameters, offering insights into the reliability of each model.

Quality parameters are represented by per-residue pLDDT scores for the native and refined AI-generated models, and z-scores for the homology models. The quartiles of those scores of all residues within each AI-predicted model are presented, along with the percentage of residues that have scores above 90 and above 70. Besides the z-score, sequence similarity and coverage, as well as the length of the predicted structures, are given as quality parameters for the homology models.

### Homology models

This data record consists of a compressed folder containing 34,613 individual PDB files, each representing a distinct homology model. Among them, 18,518 models include small molecules as ligands, and 2,829 models contain nucleic-acid molecules. Within the models, 27,932 are monomers, 4,989 were predicted as homodimers and 1,692 were predicted to be of higher oligomeric states.

### Native AI-predicted models

The compressed folder includes the AI-predicted models as they were generated by AlphaFold 2 (or downloaded from the AlphaFold database), OpenFold, and ESMFold. Each modeling type is represented by a distinct folder, named by the respective modeling type. These folders include, as individual PDB files, 41,688 AlphaFold 2 models, 41,217 OpenFold models, and 37,673 ESMFold models.

### Relaxed AI-predicted models

This data record is of the same structure as the native AI-predicted models, however, it contains the energy minimized (relaxed) structural models. The subfolders include, as individual PDB files, 41,688 relaxed AlphaFold 2 models, 41,217 relaxed OpenFold models, and 37,673 relaxed ESMFold models.

### Refined AI-predicted models

This record is of the same structure as the relaxed AI-predicted models, however, it contains the refined version of the energy minimized (relaxed) structural models. Refinement includes, as described in the Methods section, the removal of low confidence regions with a pLDDT score below 70, and filtering for models with 10 or more amino acid residues remaining. These folders include, as individual PDB files, 39,484 refined AlphaFold 2 models, 39,748 refined OpenFold models, and 31,870 refined ESMFold models.

### Relaxed AI-predicted models with ligands

The data record includes the native AI-predicted models, in which ligands were incorporated (as described in the Methods section), and which were energy minimized (relaxed) afterwards. The included subfolders contain, as individual PDB files, 15,566 ligand-containing relaxed AlphaFold 2 models, 15,487 ligand-containing relaxed OpenFold models, and 13,914 ligand-containing relaxed ESMFold models.

### Refined AI-predicted models with ligands

This data record contains the energy minimized (relaxed) ligand-containing structures which are included in the relaxed AI-predicted models with ligands, but their refined version. Refinement includes, as described in the Methods section, the removal of low confidence regions with a pLDDT score below 70, and filtering for models with 10 or more amino-acid residues remaining. After refinement, ligands which are no longer within 5 Å of any atom in the model were removed. The included subfolders contain, as individual PDB files, 14,973 ligand-containing relaxed and refined AlphaFold 2 models, 15,070 ligand-containing relaxed and refined OpenFold models, and 12,855 ligand-containing relaxed and refined ESMFold models.

## Technical Validation

### Evaluation of considered protein sequences

**Fig. 4:**
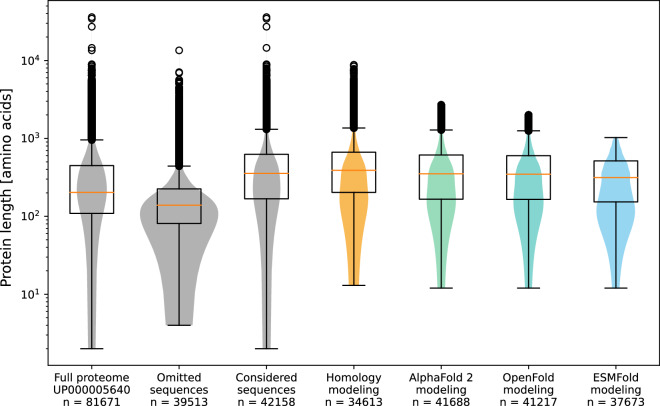
Distribution of protein sequence lengths across the modeling types. Protein length distribution for the whole UniProt reference proteome UP000005640, sequences not considered in our dataset (omitted), sequences considered in our dataset are shown, together with the length distribution of sequences which were modeled with homology modeling and the three AI-guided prediction tools AlphaFold 2 (if available, downloaded from the AlphaFold database), OpenFold and ESMFold. Boxplots are superpositioned with respective violin plots. The protein length is shown on a logarithmic scale.

Out of 81,671 protein sequences included in the UniProt reference proteome by April 2023, we considered 42,158 sequences for structural modeling. The selection included proteins whose existence had been proven on protein level and excluded protein fragments, thus consisting of full-length proteins only. The omitted sequences show shorter sequence lengths overall than the sequences considered for modeling (Fig. 4). Homology models were built for all of the considered sequences, given that the sequence suitably matched with a template deposited in the PDB, which was the case for 34,613 sequences. For the structure prediction with AI-guided tools, sequences longer than 2,700 amino acids were omitted. While AI-predicted structures always comprise the full sequence, homology models might contain only parts of the sequence, which is discussed further below in “Evaluation of homology models”. Readers can immediately assess the sequence coverage for each homology model with the provided protein information table, or by reading the respective PDB file.

### Evaluation of AI-predicted models

**Fig. 5:**
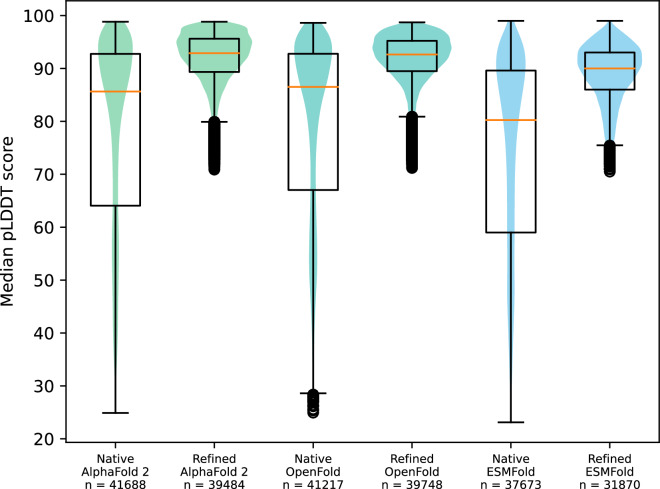
Median pLDDT score distribution of AI-predicted models. The distribution of median pLDDT scores of models built with each modeling tool (AlphaFold 2, OpenFold and ESMFold) in both their native and refined (relaxed and without low confidence regions) version, is shown as box plots superpositioned with respective violin plots.

In total 88,294 models were predicted with AI-guided tools; 32,284 AlphaFold 2 models were retrieved from existing databases^2,3^. To assess the overall quality of the models, the median pLDDT score was calculated for each model collection: AlphaFold 2, ESMFold and OpenFold; in their native and refined state (Fig. 5). Notably, refinement of AI-guided structures increased the quality of the models significantly. The individual median scores for all models, together with the pLDDT range and quartiles, as well as the distribution of residues showing a pLDDT score of >90 and >70, can be found in the provided data record “Protein_information_and_statistics.csv”. Per-residue pLDDT scores are given in the B-factor column within the respective PDB files.

### Relationship between pLDDT scores and sequence length

**Fig. 6:**
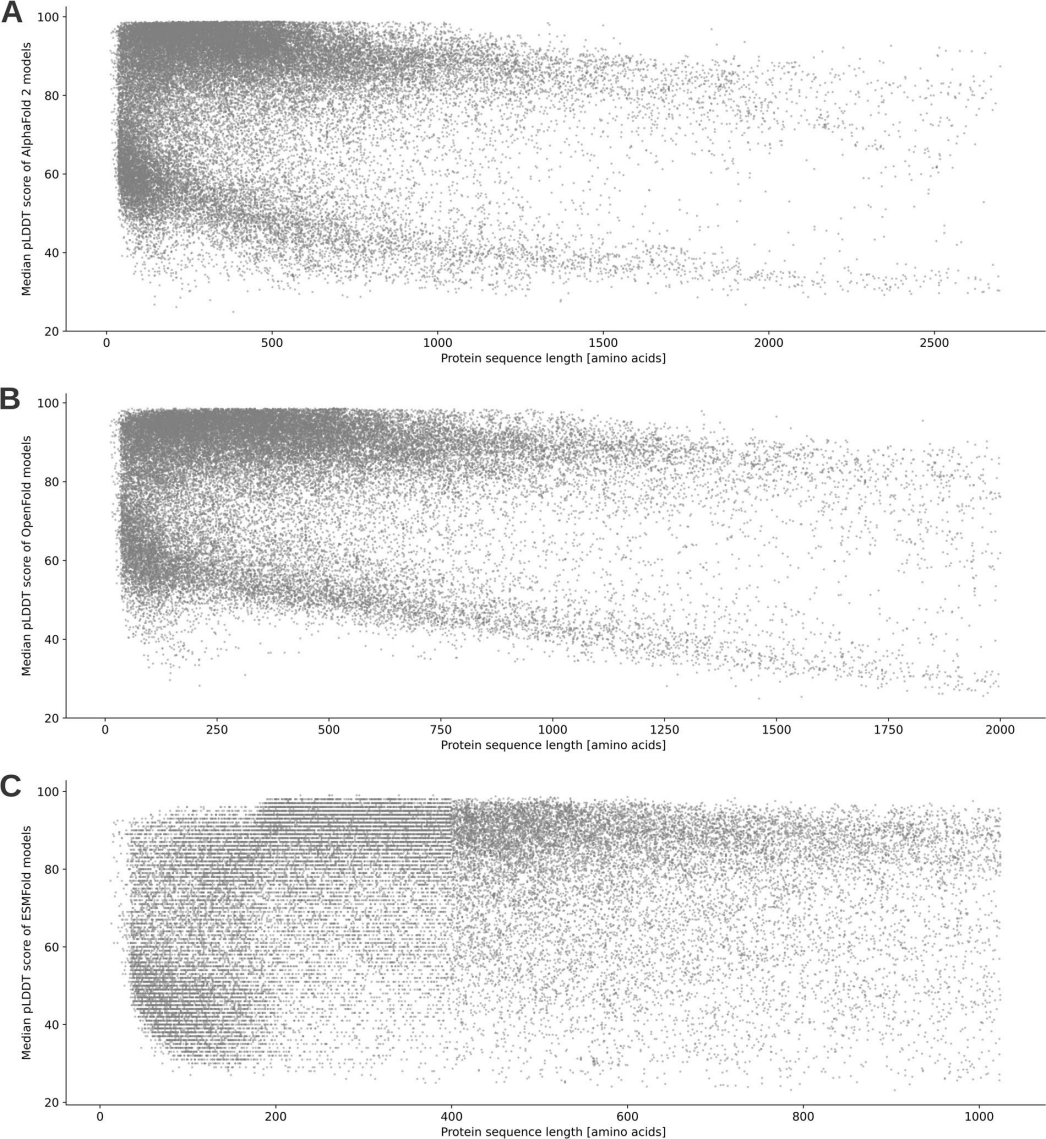
Relationship between median pLDDT scores and protein sequence length of AI-predicted models. Median pLDDT scores are plotted against the sequence length of (**A**) AlphaFold 2, (**B**) OpenFold, and (**C**) ESMFold models. Note for panel C: The median pLDDT values for proteins larger than 400 amino acids exhibit a more uniform distribution. This is because models for these proteins were generated using BioNeMo, which provides pLDDT values as percentages. In contrast, pLDDT values obtained from the Meta API, which was used for proteins up to 400 amino acids, are expressed as relative numbers. Since PDB files allow for a limited five-digit space to store pLDDT values, relative numbers (*e.g*., 0.953) contain one less digit than percentages (*e.g*., 95.32).

When assessing the relationship between the median pLDDT score of a model and its sequence length, it becomes evident that these two variables in general exhibit a relatively weak correlation (Fig. 6). Especially for AlphaFold 2 and OpenFold models (Fig. 6A,B) distinct segments within the distribution are discernible. Within this distribution, there is a segment representing high-quality models and another segment reflecting low-quality models, both of which demonstrate a decrease in quality as sequence length increases. One plausible explanation for this observed segmentation could be that for one group there is enough structural and evolutionary information available on the protein’s sequence that allows for high confidence, and for the other group, such information is only marginally available or absent. Notably, this segmentation cannot be observed for models generated with ESMFold (Fig. 6C).

### Correlation of pLDDT scores among different AI-based methods

**Fig. 7:**
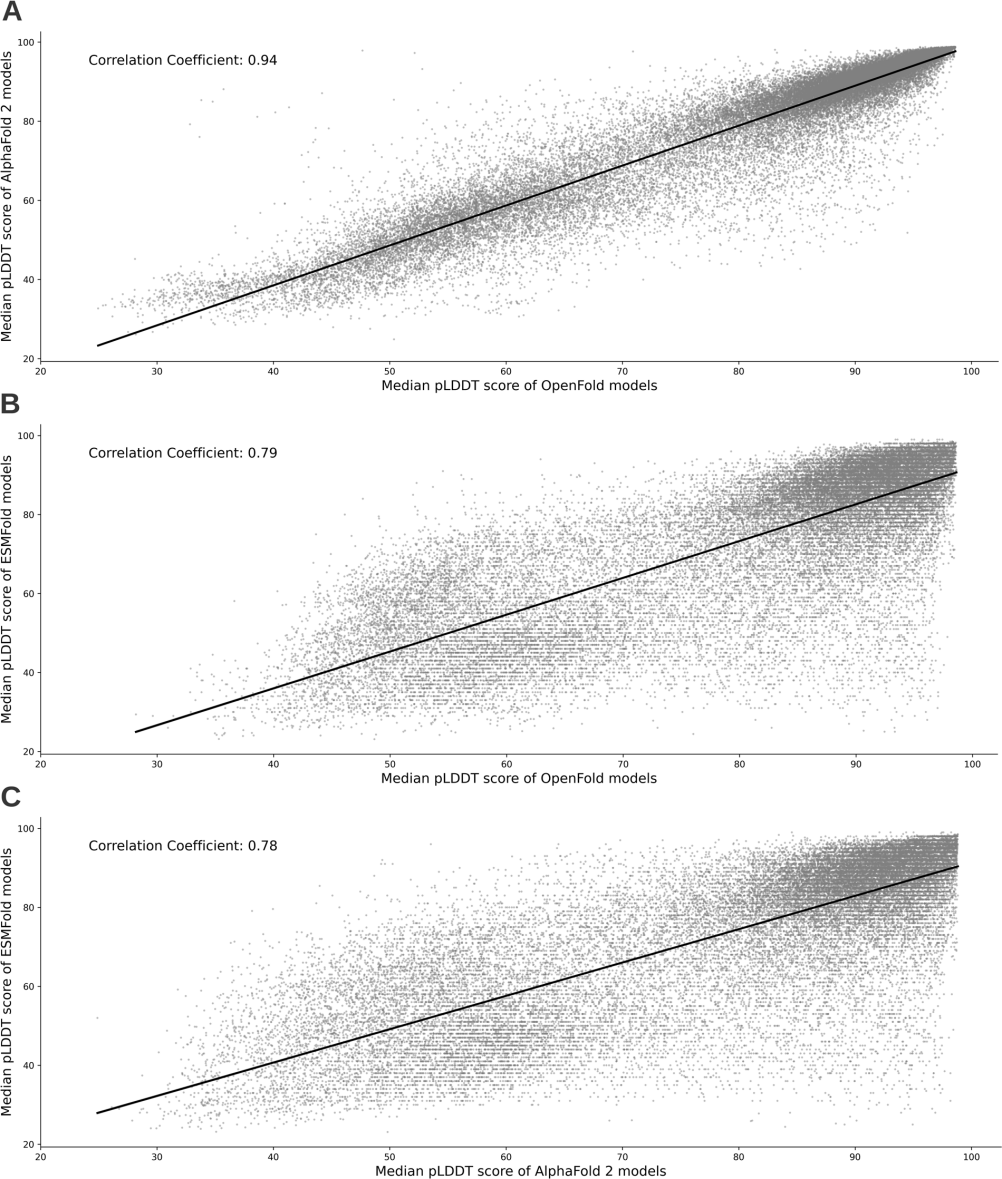
Correlation of median pLDDT scores between different AI-based prediction tools. (**A**) Median pLDDT scores of 41,217 models generated with AlphaFold 2 are plotted against the same number of models built with OpenFold. (**B**) Median pLDDT scores of 37,671 ESMFold models compared to OpenFold models. (**C**) Median pLDDT scores of 37,673 ESMFold models compared to AlphaFold 2 models. The Pearson correlation coefficients are given in the upper left corner.

Comparing the median pLDDT scores between models generated with different AI-based structure prediction tools, a high correlation in model quality can be observed, especially when comparing AlphaFold 2 and OpenFold (Fig. 7). In general, if one method predicted a model with high confidence (high median pLDDT score), also the other methods show a high median prediction confidence. Still, it is noteworthy that this is not always the case. There are exceptions for which one method generates a high-quality model and another method generates a low-quality model. These are represented in the upper-left or lower-right corner in each panel of Fig. 7 and are most prevalent when comparing with models generated by ESMFold (Fig. 7B,C).

### Evaluation of homology models

For a total of 34,613 (out of 42,158) protein sequences, a suitable template for homology modeling was found and thus 34,613 homology models were built with YASARA. The sequence identity (percentage of identical residues) and similarity (percentage of residues with similar properties) between the model sequence and the sequence of the template structure, as well as sequence coverage during the alignment process, can be used to assess the quality of homology models.

**Fig. 8:**
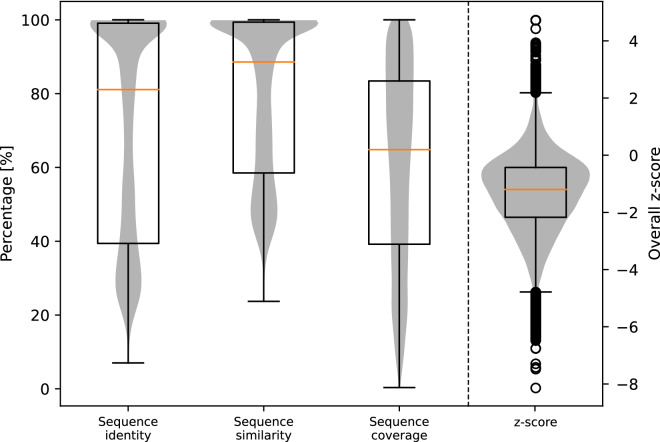
Distribution of quality parameters across 34,613 homology models. Quality parameters on sequence level (sequence identity, sequence similarity, and sequence coverage) are given in %, shown on the left y-axis. The distribution of z-scores, a quality parameter on structural level, is displayed on the right y-axis. Boxplots are superpositioned with respective violin plots.

Besides that, YASARA provides an overall z-score for each homology model, which gives quality information on a structural level. This score describes the structural similarity to the average high-resolution X-ray structure. Negative values indicate that the model is of lower quality than a high-resolution X-ray structure. Z-scores above 0 can be considered optimal, those ranging from −2 to 0 are satisfactory, while those below −2 are considered poor quality. The distribution of such quality parameters across all homology models is provided in Fig. 8. Individual values, together with oligomeric state, ligands included, and the used template from the PDB, can be derived from the provided data record “Protein_information_and_statistics.csv”.

**Fig. 9:**
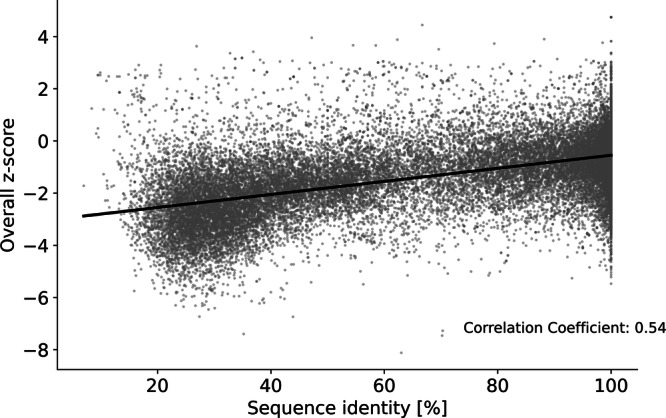
Correlation of z-score and sequence identity of 34,613 homology models. The y-axis represents the overall z-scores, while the x-axis denotes the sequence identity of these models to the template sequence. A regression line is depicted in black, revealing a Pearson correlation coefficient of 0.54.

The overall z-score of the models thereby correlates with the sequence identity (and similarity) of these models to the respective template sequence used for homology modeling (Fig. 9).

**Fig. 10:**
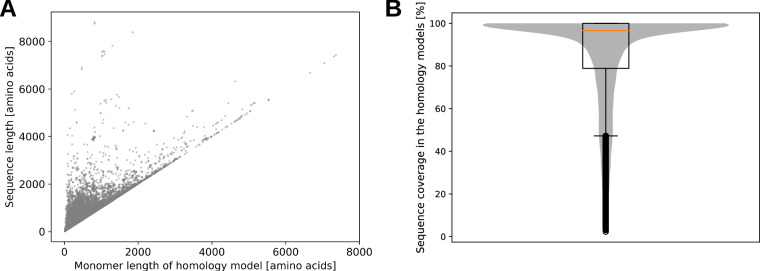
Sequence coverage in homology models. (**A**) Scatter plot comparing the length of the sequences which were submitted for homology modeling with the protein length of the resulting models. In case of multimeric homology models, the length of one monomer is shown. (**B**) Distribution of sequence coverage across 34,613 homology models. Sequence coverage was calculated by dividing the monomer length of each homology model by the length of the corresponding full protein sequence. The figure combines a boxplot and a superimposed violin plot, both derived from the same dataset.

In contrast to models generated with AI-based tools, homology models might not represent the structure for the full protein sequence, if no full-length template of suitable alignment quality is available for comparative modeling. In Fig. 10, we present an overview about the overall sequence coverage in the homology models we provide for download. A lower sequence coverage can be expected for many cases, since experimental structure determination is often not performed on the whole protein sequence. For instance, if proteins contain very dynamic parts or transmembrane regions, only the soluble and less flexible regions of the protein’s structure can be determined experimentally. Such occasions make up the vast majority of experimentally determined protein structures. Unresolved parts are then missing in the template structures used by comparative modeling and, consequently, are absent in the resulting homology models. Nevertheless, the majority of the homology models contained in the herein presented dataset comprise a high sequence coverage of above 90% (Fig. 10A,B).

## Usage Notes

### Potential usages of this dataset

The dataset of the human proteome presented herein can serve as a valuable resource for a wide range of scientific and industrial applications. In particular, this dataset enables researchers to advance machine learning approaches, using it to train models for various tasks related to protein structure and function. The availability of comprehensive structural data is fundamental for the advancement of AI-driven tools, *e.g*., RFdiffusion^16^, which is instrumental in the design of novel proteins. As the dataset provides a comprehensive structural representation of human proteins, which play a crucial role in health and disease, it further holds the potential for protein-structure-based applications in drug discovery and the development of novel therapeutic agents.

### Analyzing protein cavities with the CavitomiX PyMOL plugin

The CavitomiX PyMOL plugin (downloadable for free at https://innophore.com/cavitomix/) can be used to detect and analyze cavities, such as binding pockets and active sites, in protein structures. It further allows to compare different structural models and their cavities using crystal structures and/or AI-predicted models derived from OpenFold (powered by NVIDIA’s BioNeMo service), DeepMind’s AlphaFold and Meta’s ESMFold. If required, additional supplementary data can be obtained from the authors on request. The cavity-based Catalophore^TM^ approach has already been validated and published for the use of enzyme and drug discovery, as well as off-target identification^6–8,17^.

### Additional modeling algorithms to increase structural diversity

In this study we provide structural models of the human proteome which were built with various prediction tools to increase the diversity and to facilitate a comprehensive comparison between different modeling methods. We present structures built with tools available within NVIDIA’s BioNeMo platform (AlphaFold, OpenFold and ESMFold) and Innophore’s CavitomiX platform (homology modeling). However, we would like to point out that there are additional sophisticated tools available for structural modeling, like RoseTTAFold^18^. We encourage users to additionally consider alternative folding algorithms or molecular dynamics simulations on existing models to enhance structural diversity, if required.

### Ligand integration with AlphaFill as an alternative

As an alternative way to incorporate ligands into AI-generated models, we considered AlphaFill^19^, which gave similar, yet less ligand integration events for a selection of proteins than our method described above. To further achieve a high level of comparability between the ligand interaction sites of proteins modeled with the different structure prediction tools used in this study, we decided to instead transfer ligands from the homology models to the AI-generated models if there was sufficient structural similarity in the binding site. To increase diversity of ligand-integration events we suggest users to apply AlphaFill on AI-generated models of their proteins of interest.

### Alternative removal of low confidence regions with AlphaCutter

Notably, AlphaCutter^20^ was also considered during the refinement of native AI-predicted models for removing low-confidence parts. However, upon thorough inspection of the models, it was noted that certain extensive regions with low pLDDT values still remained. As pLDDT scores below 70 are generally considered to be of low confidence and should be observed critically, we decided to omit such regions from the refined version of the models and therefore did not employ AlphaCutter. However, we suggest that readers refine the native models we provide in our data record “Native_AI_predicted_models.zip” additionally with AlphaCutter, as it could provide different outcomes, which may be suitable for certain applications.

## Data Availability

The code used for the data preparation described herein is available on GitHub at https://github.com/innophore/structure-editing. The CavitomiX plugin for Schrodinger’s PyMOL is freely available for download at https://innophore.com/cavitomix/ and https://pymolwiki.org/index.php/CavitOmiX.
